# A Review on the Enhancement of Calcium Phosphate Cement with Biological Materials in Bone Defect Healing

**DOI:** 10.3390/polym13183075

**Published:** 2021-09-12

**Authors:** Sok Kuan Wong, Yew Hoong Wong, Kok-Yong Chin, Soelaiman Ima-Nirwana

**Affiliations:** 1 Department of Pharmacology, Faculty of Medicine, Universiti Kebangsaan Malaysia, Jalan Yaacob Latif, Bandar Tun Razak, Cheras, Kuala Lumpur 56000, Malaysia; chinkokyong@ppukm.ukm.edu.my (K.-Y.C.); imasoel@gmail.com (S.I.-N.); 2 Department of Mechanical Engineering, Faculty of Engineering, Universiti Malaya, Kuala Lumpur 50603, Malaysia

**Keywords:** biomaterials, hydroxyapatite, polysaccharide, protein, transcription factor, platelet-rich plasma

## Abstract

Calcium phosphate cement (CPC) is a promising material used in the treatment of bone defects due to its profitable features of self-setting capability, osteoconductivity, injectability, mouldability, and biocompatibility. However, the major limitations of CPC, such as the brittleness, lack of osteogenic property, and poor washout resistance, remain to be resolved. Thus, significant research effort has been committed to modify and reinforce CPC. The mixture of CPC with various biological materials, defined as the materials produced by living organisms, have been fabricated by researchers and their characteristics have been investigated in vitro and in vivo. This present review aimed to provide a comprehensive overview enabling the readers to compare the physical, mechanical, and biological properties of CPC upon the incorporation of different biological materials. By mixing the bone-related transcription factors, proteins, and/or polysaccharides with CPC, researchers have demonstrated that these combinations not only resolved the lack of mechanical strength and osteogenic effects of CPC but also further improve its own functional properties. However, exceptions were seen in CPC incorporated with certain proteins (such as elastin-like polypeptide and calcitonin gene-related peptide) as well as blood components. In conclusion, the addition of biological materials potentially improves CPC features, which vary depending on the types of materials embedded into it. The significant enhancement of CPC seen in vitro and in vivo requires further verification in human trials for its clinical application.

## 1. Introduction

Bone defect refers to the absence of bone tissue at specific anatomical positions where it is typically present, often as sequelae of trauma, infection, or removal of bone tumours [1,2]. Our skeletal system can self-repair bone defects by undergoing five main healing processes, including hematoma, inflammation, tissue granulation, callus formation, and bone remodelling. A blood clot is formed immediately after an injury providing a provisional matrix. Platelet degranulation releases inflammatory cells and mediators to activate the inflammatory stage, attracting a large number of immune, mesenchymal, and endothelial cells. The tissue granulation phase ensues, whereby the aggregated cells stimulate angiogenesis, osteoprogenitor cell proliferation, and extracellular matrix production. Subsequently, the formation of soft and hard callus improves the stability of the defect. Finally, bone remodelling occurs whereby osteoclasts resorb primary bone followed by re-establishment of bone shape and structure [3]. Typically, a bone defect has an average recovery period of six to eight weeks [4].

Autologous or artificial bone grafting is the surgical method predominantly used to rectify bone defects. The lack of readily available grafts and donor site complications often limit the clinical application of autologous bone grafts even though it is the gold standard [2]. The use of natural bone substitutes has shifted towards synthetic bone substitutes as alternatives, such as calcium phosphate-based biomaterials, to fulfil the application demands. Calcium phosphate cement (CPC) is commonly used to repair bone defects as its chemical composition is identical to the bone mineral [5]. CPC offers attractive features such as good self-setting ability, injectability, mouldability, biocompatibility, osteoconductivity, resorbability, and feasibility in controlled drug delivery [6,7]. Hence, it is the most promising and convenient injectable filler material to be used at the bone defect site. However, the critical limitations of CPC include poor mechanical strength, high brittleness, poor anti-washout behaviour, and lack of osteogenic property [8,9] (Table 1). Researchers have invested much effort to enhance their physical, mechanical, and biological performance by adding biological materials into the composition of CPC. In this context, biological materials refer to materials that are derived from living organisms. The enhancement of CPC using naturally derived materials such as bone-related transcription factors, proteins, polysaccharides, blood components, and their combinations has been demonstrated in previous scientific studies.

Herein, the reinforcement of CPC using various biological materials was summarised. The shortcomings of the current research methodology, potential research gap, and future direction have also been addressed. This review aims to discuss the broad prospects of CPC in bone tissue repair and engineering by focusing on the advantages of different biological materials in overcoming the drawbacks of CPC.

## 2. Literature Search

**Figure 1:**
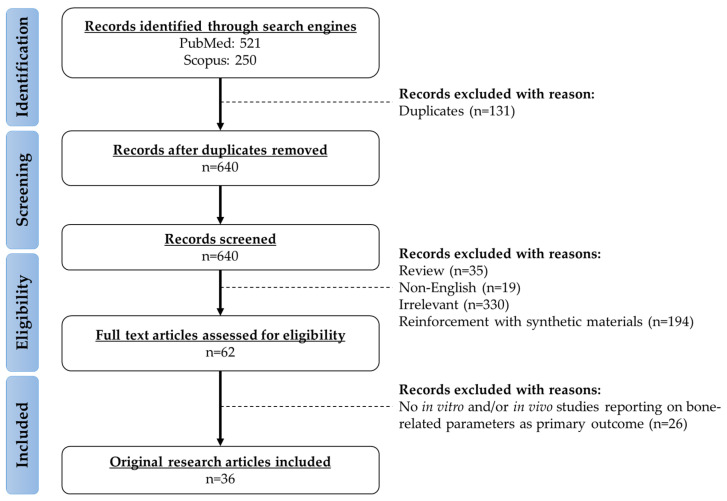
The framework for the selection of relevant studies.

A customised literature search was performed using the PubMed/MEDLINE and Scopus search engines with keywords: “(enhancement OR improvement OR reinforcement) AND (calcium phosphate cement) AND (bone OR osteoporosis OR fracture OR osteoblast OR osteoclast OR osteocyte)”. The search resulted in 521 and 250 items from the search engines respectively from inception till 15 July 2021. Duplicate articles were excluded. The titles and abstracts were initially screened based on the inclusion and exclusion criteria. The main focus of this review was to summarise the characteristics of CPC upon the incorporation of biological materials originated from living organisms. The exclusion criteria were: (a) non-English articles; (b) books, book chapters, commentaries, conference papers, letters to the editor, meta-analysis, reviews, and theses; (c) CPC reinforced with synthetic materials (such as chemicals, drugs, synthetic polymers, carbon fibres, carbon nanotubes, and metals/transition metals). Subsequently, full-text articles were screened. Original research articles reporting the effects of biological materials-enhanced CPC on bone parameters as the primary outcomes using in vitro and in vivo experimental approaches were retrieved. For example, studies that used the biological material-enhanced CPC as a scaffold for bone cells/bone progenitor cells seeding or as implant material being filled at the bone defect site of animals were included. A total of 36 related original research articles was included in this review. The framework of evidence acquisition has been summarised in Figure 1.

## 3. The Enhancement of CPC

### 3.1. Bone-Related Transcription Factors

Bone morphogenetic protein (BMP) is a group of growth factors that belong to the transforming growth factor-beta (TGF-β) superfamily. Signal transduction through the interaction between BMP and TGF-β with their receptors, i.e., bone morphogenetic protein receptor (BMPR) and transforming growth factor-beta receptor (TGF-βR), results in the phosphorylation of SMAD proteins and transcription of Runx-2 (a master regulator of osteoblastogenesis). Apart from the potent osteoinductive properties, BMP and TGF-β have other non-skeletal actions. For example, they enhance the development of new blood vessels and restoration of microcirculation by promoting the synthesis of pro-angiogenic factors that provide oxygen and nutrients to the injured site, stimulate the formation of tip cells and sprouting, as well as migration and proliferation of endothelial cells [10,11]. Hence, BMP and TGF-β have been potentially used as a new adjunct to graft materials for bone regeneration (Table 2).

An earlier in vitro study pointed out that the addition of recombinant human TGF-β1 (rhTGF-β1) to CPC increased ALP activity in two cell populations, the pre-osteoblastic and osteoblastic cells isolated from adult rat long bones [12]. The incorporation of recombinant human BMP-2 (rhBMP-2) increased the compressive strength of the CPC scaffold [14]. A study by Zhang et al. demonstrated that the viability of myoblastic precursor cells was promoted and the expression of osteogenic genes (including alkaline phosphatase (ALP), type I collagen (COL1), osteocalcin (OCN), and Runt-related transcription factor 2 (Runx-2)) were elevated when seeded on CPC/rhBMP-2 scaffold. Using New Zealand white rabbits as an animal model of femoral bone defect, the same group of investigators reported that the residue of material (CPC scaffold with rhBMP-2) was decreased while bone ingrowth and newly formed bone area were increased at the defect site after eight weeks of implantation [13]. Another group of researchers implanted a CPC scaffold loaded with rhBMP-2 at the defect area in the middle radius of white rabbits. Qualitative assessment of bone defect using radiograph and micro-computed tomography indicated that the defect area was filled by the implanted material and callus formation was observable as early as two weeks after implantation. The animals with defect areas implanted with CPC and rhBMP-2 also had higher bone mineral content (BMC), bone mineral density (BMD), and maximum load compared to those animals without any implants [14]. Recently, Tao et al. investigated the effects of strontium-modified CPC dripped with a single-dose local administration of BMP-2 on healing bone defects in osteoporotic rats induced by ovariectomy. The improvement of bone defect healing was noted in animals eight weeks after implantation with strontium-modified CPC in the presence of BMP-2 local administration, evidenced by denser bone microstructure, greater newly formed bone, decreased percentage of remaining biomaterials, and higher ultimate load [15].

Overall, the scientific evidence showed that BMP-2 enhanced the maximum compressive strength of CPC. Several in vitro and in vivo studies consistently supported that BMP-2 stimulated osteogenic differentiation leading to enhanced bone formation and regeneration in animals with cavity defects.

### 3.2. Proteins

Proteins, such as silk fibroin and amino acids, have been used as enhancers to improve the mechanical properties of CPC (Table 3). Silk fibroin is a natural protein biopolymer found abundantly in silkworms, *Bombyx mori*. Silk has been traditionally used as a luxury raw material in the textile industry. The advancement in technology revealed that silk fibroin is a biomaterial with excellent biocompatibility and natural strength, suggesting its wide application for drug delivery, vascular tissue regeneration, wound treatment, and bone tissue scaffold [16]. The reinforcement of CPC by alkaline-treated silk fibroin was developed by Hu et al. and the features were evaluated. The compressive strength was improved, setting time was shorter, no collapse was observed, and injectability was increased in cement after adding silk fibroin solution. Ex vivo analysis on sheep vertebrae implanted with CPC containing silk fibroin showed increased mechanical strength and stiffness. The MC3T3-E1 cells seeded on silk fibroin/CPC showed increased proliferation and normal cell morphology [17].

Amino acids are organic compounds consisting of two functional groups (amino (–NH_2_) and carboxyl (–COOH)) and a side chain. Lysine is an essential amino acid that must be obtained from the diet. It is important for the synthesis of protein, hormone, enzyme, collagen, elastin, and absorption of intestinal calcium, suggesting its role in maintaining skeletal homeostasis [30]. Thus, lysine has been incorporated into CPC to promote osteogenic activity. A recent study examined the characteristics of CPC with lysine as well as the expression of osteogenic markers in bone mesenchymal stem cells seeded on lysine-incorporated CPC. Results showed that the presence of lysine in CPC improved mechanical strength and apparent porosity in a lysine-content-dependent manner [18]. The pore size of a scaffold regulates osteogenesis as it affects bone cell adhesion, proliferation, and distribution. Scaffold with high porosity allows adequate diffusion of nutrients, bone cell migration, and provides sufficient surface area for cell-biomaterial interactions [31]. In this context, the scaffold porosity should be balanced with the mechanical needs for bone tissue at the defect site as mechanical strength decreases with increasing porosity. The in vitro findings from a study by Shi et al. showed that the ALP activity and osteogenic protein expression were increased after seven days of culturing bone mesenchymal stem cells on CPC incorporated with lysine [18].

A tripeptide motif, consisting of amino acids arginine, glycine, and aspartate, is initially identified as an amino acid sequence that facilitates cell attachment. It is present in various proteins of the extracellular matrix, including fibronectin, vitronectin, and fibrinogen. This tripeptide motif has a strong affinity towards integrins, the transmembrane glycoproteins involved in anchoring cells to the extracellular matrix [32]. It is known that integrins are expressed in various types of cells including leukocytes [33], macrophages [34], endothelial cells [35], and bone cells [36,37]. Recruitment of these cells through recognition of the arginine-glycine-aspartate motif on biomaterial surface is important for tissue regeneration, including the regeneration of bone defects. It is worth mentioning that the addition of peptide sequences arginine-glycine-aspartate motif into CPC did not compromise the setting time, flexural strength, elastic modulus, and work to fracture of CPC [19,20]. The adding of arginine-glycine-aspartate improved human umbilical cord mesenchymal stem cell attachment. Faster cell proliferation, increased cell number, higher osteogenic gene expression as well as higher mineral concentration were noted, indicating osteogenic differentiation of human umbilical cord mesenchymal stem cells [19,20].

N-acetyl cysteine (NAC) is an acetylated product of cysteine, a semi-essential amino acid naturally found in the human body. It is a known medication used to treat overdose of paracetamol through its action as the precursor of glutathione to conjugate and deactivate toxic *N*-acetyl-*p*-benzoquinone imine metabolised from paracetamol [38]. The anti-oxidative ability of NAC suggests its potential in promoting bone health and osteoinduction. Recently, Feng et al. prepared a CPC composite by mixing NAC loaded silk fibroin solution with α-tricalcium phosphate (α-TCP) and tested the osteogenic properties ex vivo, in vivo, and in vitro. In the ex vivo experiment, a bone defect (6 mm) was created by surgical drilling through the vertebral body perpendicular to the sagittal plane of the excised sheep lumbar vertebra and filled with silk fibroin-NAC/α-TCP. Higher compressive strength and stiffness were detected in the lumbar vertebra after being filled with silk fibroin-NAC/α-TCP. Maximum force, BV/TV, and rate of material degradation were increased in the rats subjected to surgical drill to create a critical-size bone defect at femoral condyle which was filled with silk fibroin-NAC/α-TCP. An in vitro study using rat bone marrow mesenchymal stromal cells showed higher ALP activity, mineralisation, and osteogenic genes expression after being seeded onto cement with the addition of silk fibroin and NAC. These osteogenic actions were inhibited after the addition of Dickkopf-related protein-1 (DKK-1, a known Wnt inhibitor) in the culture, thus suggesting the postulated signalling pathway involved in the osteogenic enhancement was mediated through the activation of Wnt/β-catenin [21].

Collagen consists of amino acids that are bound together to form a triple collagen helix, which has a main structural function in connective tissues, such as bone, cartilage, tendon, ligament, and skin. Collagen has been primarily used for cosmetic, nutraceutical, pharmaceutical, and medical applications. Previous investigations indicated that dietary hydrolysed collagen increased osteoblast activity, leading to improved bone architecture and biomechanical resistance in osteoporotic mice [39]. In view of the potential skeletal-promoting effects of collagen, its combination with CPC had better bone repair effects mainly due to the biomimetic properties of CPC that resembled the composition of natural bone. Collagen had been added into composite scaffolds to enhance osteoblast proliferation and load-bearing capacity [22]. The cell attachment, proliferation, and osteogenic differentiation of human umbilical cord mesenchymal stem cells on the collagen-CPC scaffold were investigated. Collagen fibres in CPC improved the mechanical properties. The presence of mineral nodules, increase in an extracellular matrix formation, cell numbers, and higher osteogenic expression were noted in human umbilical cord mesenchymal stem cells cultured on CPC with collagen than those on CPC without collagen [22]. Likewise, the presence of collagen microsphere in injectable cement paste facilitated new bone formation after it was implanted at a defect site in the femoral condyles of female New Zealand rabbits [23].

Gelatine is a colourless, tasteless, odourless, and translucent protein product from animal-derived collagen. It dissolves in warm water and forms a jelly-like texture at lower temperatures, providing a binding force [40]. Gelatine has been used in food and cosmetic applications, acting as stabiliser and thickener in foods or texturiser in conditioners and moisturisers. Gelatine is less antigenic than collagen and binds well to tricalcium phosphate, suggesting that it is an excellent material for bone replacement [41]. Two groups of researchers synthesised an injectable homogeneous paste comprising of CPC with or without gelatine. The initial and final setting time was decreased whereas the mechanical strength was increased by adding gelatine into CPC. In vitro experiment showed that the cell number, proliferation, ALP activity, type 1 procollagen, and TGF-β1 level were induced in G-292 and MG63 osteoblastic cells when seeded on gelatine-containing CPC as compared to those without gelatine additives [24,25].

The elastin-like polypeptide is a protein-based polypeptide that consists of valine-proline-glycine-Xaa-glycine (Val-Pro-Gly-Xaa-Gly) pentapeptide repeats, in which Xaa can be a variable naturally occurring amino acid, except for proline [42]. It has been developed using genetic engineering technology with excellent properties of biocompatibility, non-immunogenic, non-toxic, and controllable degradation [26]. The addition of elastin-like polypeptide into CPC increased micro-hardness, compressive strength, washout resistance as well as initial and final setting time. The microstructure of the biomaterial was denser with fewer pores, larger crystallites, and fewer sharp edges, indicating the ability of the elastin-like polypeptide to stabilise CPC. Mouse embryonic fibroblast (NIH3T3) cells remained viable and displayed normal cell morphology, spreading pattern, cell distribution, and no nuclear condensation after being cultured on CPC supplemented with elastin-like polypeptide [26].

Calcitonin gene-related peptide (CGRP) is an amino acid peptide mainly produced in both central and peripheral neurons. It is primarily released from innervating sensory fibres and possesses potent vasodilator activity. Thus, it is implicated in the pathophysiological conditions involving the cardiovascular system, wound healing, and nociception [43]. The role of CGRP on bone remodelling and regeneration has also been reported within in vivo studies. CGRP enhanced blood vessel formation and bone regeneration in a rat model of distraction osteogenesis [44]. Mice with CGRP deficiency subjected to a femoral osteotomy had reduced bone-forming osteoblast number, higher rate of incomplete callus bridging, and fracture non-union [45]. The enrichment of CPC with CGRP did not alter pore size distribution and compressive strength of CPC, but this material increased cell proliferation, ALP activity, expression of BMP-2, osteonectin, and Runx-2 in rat bone marrow mesenchymal stem cells [27].

Bone sialoprotein is a non-collagenous protein synthesised by mineralising connective tissues, such as bone, dentin, cementum, and calcified cartilage tissues [46]. It regulates hydroxyapatite crystal formation in bones and teeth as well as contains an arginine-glycine-aspartate sequence to attract cells and support cell adherence [47,48]. As a result, higher numbers of osteoblast cells grew on the bone sialoprotein-functionalised CPC scaffolds compared to the untreated CPC scaffolds. However, significant changes in the expression of osteogenic genes were not detected among the scaffolds coated with and without bone sialoprotein [28]. Subsequently, the same group of investigators investigated the effects of bone sialoprotein-coated CPC on bone formation in a femoral defect rat model. Their findings indicated that the implantation of the bone sialoprotein-CPC scaffold into the defect site caused minimal changes in bone regeneration and ingrowth after 8 weeks [29].

In general, most of the proteins improved washout resistance, injectability, compressive strength, and reduced the setting time of CPC. Exceptions were seen in the arginine-glycine-aspartate motif, elastin-like polypeptide, and CGRP. The tripeptide motif was insufficient in enhancing the biomechanical strength of CPC. Elastin-like polypeptide delayed the setting reaction of CPC, mainly attributed to their interaction which decreased the hydration of CPC. Meanwhile, the enrichment of CGRP did not affect the compressive strength and pore size distribution of CPC. These characteristics might be the challenges for its clinical applications. Furthermore, different types of proteins have different effects on pore size distribution. For instance, lysine increased the apparent porosity of CPC whereas elastin-like polypeptide-supplemented CPC had a smaller pore size indicating denser microstructure. In terms of biological performance, favourable improvements were noted by supplementing proteins into CPC with increased cell viability and osteogenic activity. However, the coating of bone sialoprotein on CPC supported cell attachment, but a lesser tendency towards osteogenic gene upregulation and bone formation.

### 3.3. Polysaccharides

Polysaccharides, including cellulose, chitosan, hyaluronic acid, and alginate, have received much attention as biomaterials for bone regeneration (Table 4). Cellulose is the most abundant organic biopolymer with the formula of (C_6_H_10_O_5_)_n_. It is a polysaccharide that consists of linear chains of repeated β-D-glucopyranose units linked by β-1,4-glycosidic bonds. Cellulose can be derived from plants, algae, and bacteria. Cellulose secreted by gram-negative bacteria has a similar molecular structure but exhibits several advantages over plant-derived cellulose, such as high purity, tensile strength, and large surface area. These intrinsic characteristics make it an ideal biomaterial in various medical applications, such as wound dressings, drug delivery, skeletal and cartilage substitutes [49]. A study by Zhang et al. reported that the bacterial cellulose-reinforced CPC composite had higher thermal stability and compressive strength than CPC. The composite also promoted cell growth, cell proliferation and displayed higher cell survival than CPC when seeded with MC3T3-E1 cells, indicating improved biocompatibility [50].

Chitosan is a natural linear polysaccharide derived from chitin, the second most abundant polysaccharide after cellulose. It can be extracted from the exoskeleton of insects, the hard outer skeleton of shellfish (including crab, lobster, and shrimp), and the fungi cell wall. Chitosan is a versatile biomaterial due to non-antigenicity, biocompatibility, biodegradability, non-toxicity, good adsorption, and anti-bacterial potency [57]. However, its application is limited by its insolubility in neutral and aqueous solutions [58]. The addition of chitosan into other materials, such as ceramics, composites, and hydrogels, broadens its application as a scaffold component with new functional properties. The differences in elastic modulus, compressive and flexural strength between CPC reinforced with chitosan and those without chitosan were significant, with the former group higher than the latter group [52,53]. In vitro, the ALP activity was higher in rat bone marrow mesenchymal stem cells or osteoblastic cells cultured on CPC-chitosan than in CPC alone [51,52]. Radiographic examination detected the increase in new bone callus formation and reduction in material debris after the filling of chitosan-reinforced CPC into periosteum bone defect at the left canine radius for 20 weeks compared to those filled with CPC without chitosan [53].

Hyaluronic acid, also known as hyaluronan, is a naturally occurring linear glycosaminoglycan that is widely distributed in the connective tissues including skin, synovial fluid, vitreous, and cartilage. It exhibits properties of high viscosity, elasticity, negative charge, biocompatibility, biodegradability, and non-immunogenicity [59]. Hyaluronic acid has been widely employed in the applications of drug delivery [60], cosmetics [61], cancer diagnosis [62], wound healing [63], orthopaedics [64], tissue engineering [65], and tissue regeneration [66]. The addition of hyaluronic acid into CPC significantly increased the compressive strength [54]. Better ALP activity was observed in osteoblastic cells cultured on CPC with hyaluronic acid than CPC alone [51]. Cui et al. reported higher protein expression of ALP, OCN, and BMP-2 in human bone marrow mesenchymal stromal cells after being cultured with hyaluronic acid-containing CPC relative to those without hyaluronic acid. The material has also been tested by implanting it into adult female rats with a bone defect (2 mm diameter × 2 mm depth) at the metaphyseal region of the medial tibia. The findings of this study indicated denser calcified new bone, higher mineralisation, improved bone microarchitecture, and stronger positive expression of osteogenic markers in animals receiving hyaluronic acid-CPC implant than those receiving only CPC implant [54].

Another natural water-soluble polysaccharide, alginate, can be isolated from the cell wall of brown seaweed or algae. It has multifaceted roles in the biomedical and pharmaceutical fields due to its biocompatibility, non-immunogenicity, non-toxicity, low cost, and mild gelation. Alginate is commonly used in hydrogel form, providing a three-dimensional cross-linked network for migration of cells, delivery of bioactive agents, and stability of structure [67]. Several reports supported the role of alginate in improving the injectability, cohesion, strength, and elasticity of CPC without affecting the hardening rate. For instance, in vitro cell tests demonstrated that alginate-added CPC did not harm the viability of human osteoblast-like cells and human umbilical cord mesenchymal stem cells [55,56]. The differentiation capability of human umbilical cord mesenchymal stem cells into osteogenic lineage was enhanced with elevated ALP, OCN, COL1, and OSX expression [56].

Based on the collated evidence, polysaccharides enhanced CPC physically, mechanically, and biologically. The injectability, thermal stability, cohesion, mechanical strength, osteogenic activity, and bone formation were promoted without affecting the setting time of CPC.

### 3.4. Blood Components

Whole blood is a specialised body fluid with four main components, namely plasma, red blood cells, white blood cells, and platelets. Blood has multiple functions, including transporting oxygen and nutrients, forming blood clots to prevent excessive blood loss, carrying cells and antibodies to fight infection, bringing waste products to kidneys and livers for excretion, and maintaining the body’s pH and temperature. Considering that hematoma formation occurs as the first stage of bone healing after trauma, the addition of blood components might enhance the osteoinductive properties of CPC (Table 5). In the study conducted by Mellier et al., autologous whole blood stabilised by sodium citrate was used as the liquid phase for CPC paste formation. The CPC-blood composite was implanted into a cylindrical osseous critical-sized defect at the distal femoral end of female New Zealand white rabbits. The animals receiving CPC/blood composite exhibited higher material degradation and new bone formation 12 weeks after implantation than those receiving CPC only. The physical characteristics of CPC after incorporation with whole blood showed an increase in setting time, decrease in stiffness, and no change in compressive strength [68].

Platelet-rich plasma, also known as autologous conditioned plasma, is plasma with concentrated platelets obtained from centrifugation or gravity filtration of autologous blood. It receives much attention in recent years for its application in different medical fields. The widespread clinical uses of platelet-rich plasma are mainly attributed to several factors: (a) the large amount of growth factors and various proteins stimulates the tissue healing process; (b) the neovascularisation ability provides blood supply and nutrients essential for cell proliferation, differentiation, and tissue regeneration; (c) the autologous characteristic prevents the risk of crossed contamination, disease transmission, and immune reactions [72]. The performance of CPC was enhanced by platelet-rich plasma additives, with enhanced viscosity, no disintegration of paste consistency, and decreased setting time. MC3T3-E1 cells seeded on the brushite-based CPC in the presence of platelet-rich plasma demonstrated increased cell density, homogenous cellular distribution, and higher cell-to-cement interaction. New Zealand white rabbits implanted with platelet-rich plasma-enhanced CPC had higher BV/TV and degradation rate after four weeks. Histological findings showed the recruitment of fibrous tissues at the early stage of implantation, which later disappeared to facilitate bone formation [69]. In female ovariectomised rats with a cavity-like defect at the distal 1/3 of the caudal vertebral body, the augmentation of CPC with platelet-rich plasma as filler material improved trabecular bone microstructure, BMD, new bone formation, and osteogenesis grade as compared to those implanted with CPC only [1]. Likewise, the enhancement of osteogenic properties of CPC after the addition of platelet-rich plasma was reported by another group of researchers. The ALP production of bone marrow mesenchymal stem cells from BALB/c mice grown on CPC containing platelet-rich plasma composite surface was higher than CPC only. The in vivo rabbit model of bone defects also indicated a breakdown of bulk dense CPC/platelet-rich plasma implants into pieces with higher trabecular bone tissues around the implant site over time [70].

Fibrin glue (or sealant) is a blood-derived biological tissue adhesive product consisting of a fibrinogen component and a thrombin component, which induces the coagulation cascade. It can be prepared from the plasma of individual volunteers with a low yield of fibrinogen or produced commercially using pools of plasma with a high concentration of fibrinogen [73]. Fibrin glue has been widely used in surgical procedures for wound closure, tissue repair, wound healing, and prevention of leakage or bleeding [74]. Dong et al. introduced fibrin glue purified from the blood into CPC powder to be inserted into the rabbit femoral defect model. The compressive strength and elastic modulus of the rabbit femur filled with CPC-fibrin glue were higher than those filled with CPC only. Analyses from micro-computed tomography and histological examination showed a greater amount of newly formed bone in the group provided with CPC-fibrin glue implants compared to the animals with CPC implants [71]. However, the application of human-derived fibrin glue is limited by the possibility of blood-borne disease transmission [75] and antibodies development [76]. Recently, researchers have attempted to establish fibrin glue extracted from snake venom attributed to its favourable properties for not causing adverse side effects, not having human blood components, not transmitting diseases, and has the good adhesive ability [77]. Thus, the use of heterologous snake venom fibrin glue as a potential candidate to enhance the physical, mechanical, and biological characteristics of CPC should be tested. The effects of snake venom fibrin glue in promoting bone regeneration in cancellous bone defects requires investigation from scientific studies.

The combination of CPC with blood components can influence the biological properties of the composites, particularly the increases in osteogenic proliferation and cell-to-cement interaction with no cytotoxic effect. In terms of physical performance, although the whole blood prolonged the setting time of the composite, the authors claimed that it did not limit its practical use in bone surgery as the cohesiveness was higher than the blood-free analogue. The ability of blood clot formation caused by whole blood prevented disaggregation of the composite by body fluids at the defect site [68]. Newer advancements in using platelet-rich plasma may be a better option as there was no disintegration and faster setting reaction during implantation, which can withstand segregation and stabilise the defect site, followed by its ability to degrade after a period of time to allow the growth of new bone tissue. On the other hand, fibrin glue may be superior in enhancing the mechanical properties of CPC as compared to other blood components. Improvements are warranted to enhance the biomechanical strength of CPC incorporated with blood composite and platelet-rich plasma.

### 3.5. Combination of Biological Enhancers

The blend of two different enhancers could be an idea to improve the features of CPC further. BMP-2 combined with other enhancers (such as collagen, gelatine, and silk fibroin) has also been previously utilised to reinforce the CPC scaffold (Table 6A). Based on the above discussion, supplementing the individual enhancer (either BMP-2 or collagen) into CPC conferred better biomechanical strength [14,22]. Lee et al. developed an injectable CPC with collagen and BMP-2 loading, which displayed the characteristics of decreased setting time with no disintegration apart from increased mechanical strength relative to the CPC without enhancement [78]. Findings from this study suggested that the combination of BMP-2 and collagen was superior in reinforcing CPC than its individual component. Mouse pre-osteoblast (MC3T3-E1) cells cultured on the combined material displayed rapid cell viability, cell proliferation, and cell spreading. In addition, qualitative micro-computed tomography scanning images showed defect integration, new bone tissue formation, and material degradation of CPC loaded with BMP-2 and collagen after 4 weeks of implantation at the cylindrical defect created on the parietal part of the femur of New Zealand white rabbits [78]. Using bigger animals as models, female goats were subjected to ovariectomy to induce osteoporosis and bone defect at the lumbar vertebrae, which was filled with CPC containing BMP-2 loaded gelatine microspheres. The implanted vertebral bone of the animals had greater mechanical strength and bone mineralisation rate after 45 days than the untreated group [79]. In the ovine model of interbody defect created at the midpoint of disc space, the site filled with CPC/silk fibroin/rhBMP-2 composite exhibited higher stiffness and bone volume, which were on par with those implanted with autograft. Lower material residue volume was also detected in CPC reinforced with silk fibroin and rhBMP-2 compared to the material without rhBMP-2 [80].

Proteins and polysaccharides have been combined and incorporated into CPC to enhance osteogenic effects and mechanical properties (Table 6B). The addition of peptide sequences arginine-glycine-aspartate motif into chitosan-containing CPC enhanced flexural strength and work of fracture but did not have any effect on elastic modulus [81,82]. Compared to CPC reinforced with arginine-glycine-aspartate motif alone, which did not cause any change in biomechanical strength [19,20], the addition of chitosan further improved the mechanical strength of CPC incorporated with the arginine-glycine-aspartate motif. Two groups of researchers investigated the osteogenic proliferation and differentiation of human embryonic stem cell-derived mesenchymal stem cells and mouse pluripotent C3H10T1/2(C3) cells on CPC with the addition of chitosan immobilised with peptide sequences arginine-glycine-aspartate motif. Their results demonstrated higher cell number, proliferation, differentiation, and mineralisation [81,82]. A similar biomaterial was also used as a filler for a 3 mm diameter and 6 mm depth bone cavity at femoral condyles of New Zealand white rabbits. The implanted material underwent degradation in the femur cavity with lesser residual found in animals receiving CPC-chitosan with arginine-glycine-aspartate compared to those receiving CPC-chitosan [82].

Two different types of polysaccharides were also combined and introduced into CPC, in which the bone regeneration properties have been tested in vivo (Table 6C). The mixture of chitosan-alginate complex and CPC produced a gel-like matrix, with reduced setting time, no disintegration, and increased compressive strength [83]. This combination reduced the time for CPC to harden, which was not seen in CPC supplemented with alginate alone. Following implantation of the chitosan-alginate complex in vivo to a cylindrical defect at the femoral head of male New Zealand white rabbits, an increased amount of newly formed bone with less implant remaining and denser mineralisation were noted [83]. A similar biomaterial was created by another group of researchers, consisting of CPC and alginate-chitosan microencapsulated with mouse osteoblast MC3T3-E1 cells. They researched the osteogenic potential of this complex by injecting it into the dorsal subcutaneous area of BALB/c nude mice. The results revealed higher lamellar bone-like mineral structure, newly formed collagen, mineralisation rate, and less scaffold material remaining in the animals implanted with CPC containing alginate-chitosan and MC3T3-E1 cells compared to those implanted with CPC alone [84].

Taken together, different combinations of biological enhancers may not necessarily improve the limitations of CPC. Positive improvements of CPC were seen in the composites with the presence of BMP-2 and other biological enhancers as well as two types of polysaccharides, but not in the mixture of protein and polysaccharides. However, there is only a paucity of studies available to support these findings. Further studies are recommended to validate this hypothesis.

## 4. Perspectives

Calcium phosphate cement can self-harden and incorporate various components (such as drugs and biological materials) owing to its intrinsic porosity without affecting its functions. In this review, the scientific evidence pointed out that the incorporation of biological materials has great potential to resolve the limitations and further enhance the characteristics of CPC. Although some biological materials may be insufficient to reinforce CPC, their incorporation did not cause any deterioration on the beneficial features of CPC. Most of these biological materials increased the physical, mechanical, and biological performance resolving the brittleness, lack of osteogenic properties, and poor anti-washout of CPC, except for incorporating the elastin-like polypeptide, CGRP, bone sialoprotein, and blood components. A summary of the effects of biological materials in improving the characteristics of CPC has been provided (Table 7).

The limitation of current evidence has been acknowledged. The evaluation of biological materials-enhanced CPC in most in vivo bone defect animal models was performed radiologically and histologically with qualitative but not quantitative analysis. The quantifications of bone microstructural parameters and bone cells at multiple time points allow the observation of dynamic bone tissue healing and regeneration process at the defect site. Apart from that, paucity in the number of original studies needs to be addressed. The characteristics of CPC enhanced with single materials and those enhanced with multiple materials could not be compared directly due to the lack of original studies. Therefore, the comparison was drawn from different studies with possible different experiment conditions and the readers should read with caution.

Several recommendations for future studies are suggested. Firstly, various plant extracts and their isolated bioactive compounds have been extensively demonstrated by researchers to exert potent bone-protecting properties [85,86,87,88,89,90], which could be introduced into CPC to improve its physical, mechanical, and osteogenic effects. Secondly, a three-dimensional osteoblast-osteoclast co-culture system mimicking the skeletal microenvironment in humans could be used to investigate the properties of CPC after the reinforcement using biological materials [91,92]. The positive outcomes of biological material-enhanced CPC in promoting bone healing and tissue regeneration observed in the in vitro and in vivo evidence await further validation on its performance for clinical applications.

## 5. Conclusions

In summary, the current evidence demonstrated the potential enhancement of CPC properties by introducing biological materials. However, the addition of different materials may result in different degrees of improvement in CPC. The desired bone graft substitutes should display the characteristics of injectable, cohesive, resorbable, self-hardening, and high mechanical strength that allows rapid cell invasion, cell proliferation, and osteogenic activity, which may be a major challenge to designing a single bone graft substitute that fulfils all the criteria. From the articles reviewed here, polysaccharides are the best biological additives for CPC as they fulfil most of the criteria of an excellent bone graft. With the advancement of new technology, the development of tailor-made bone substitutes by incorporation of bioactive substances into CPC is the way forward to meet the demands for use in research and in the clinical setting.

## Figures and Tables

**Table 1:** The characteristics of ideal bone cement.

Characteristics	Criteria for an Ideal Bone Cement
Self-setting ability	The bone cement should harden in situ, forming solid hydroxyapatite.
Injectability	The bone cement could be pushed out through a syringe without separation of the liquid and powder that composed it.
Mouldability	The bone cement could be moulded according to the shape of a bone cavity
Biocompatibility	The bone cement should not cause any local or systemic adverse response.
Osteoconductivity	The bone cement should encourage host bone cells, capillaries, and tissue to move into it to facilitate bone repair.
Resorbability	The bone cement should be resorbable by the body.
Feasibility in controlled drug delivery	The bone cement could be designed to deliver a drug at a predetermined rate.
Mechanical strength	The bone cement could withstand application of force without failure and deformation.
Brittleness	The bone cement should not be fractured easily when subjected to stress.
Anti-washout	The bone cement should be resistant to segregation under washing action.
Osteogenic property	The bone cement should encourage new bone formation by osteoblasts.

**Table 2:** The bone protecting effects of CPC enhanced by BMP-2 and TGF-β.

Enhancer	Characteristics of Enhanced CPC	Type of Study	Type of Cell, Sample, and Animal Model	Outcomes Observed in Animals	Reference
rhTGF-β1	-	In vitro	Pre-osteoblastic and osteoblastic cells obtained from collagenase-treated fragments of adult rat long bones	Cell differentiation: ↑, ALP: ↑	[12]
rhBMP-2	-	In vitro	Myoblastic precursor cells	ALP: ↑, COL1: ↑, OCN: ↑, Runx-2: ↑	[13]
		In vivo	Bone defect at femur of female New Zealand rabbits	BV/TV: ↑, residue of material: ↓, newly formed bone area: ↑	
rhBMP-2	Maximum compressive strength: ↑	In vivo	Critical defect at the middle of the radius of male New Zealand rabbits	BMC: ↑, BMD: ↑, bone formation: ↑, bone regeneration: ↑, maximum load: ↑	[14]
BMP-2	-	In vivo	Bone defect at femoral metaphysis of ovariectomised rats	BV/TV: ↑, Tb.N: ↑, Tb.Th: ↑, Tb.Sp: ↓, newly formed bone: ↑, percentage of remaining biomaterial: ↓, ultimate load: ↑	[15]

Abbreviations: ALP, alkaline phosphatase; BMC, bone mineral content; BMD, bone mineral density; BV/TV: bone volume/total volume; COL1, type I collagen; CPC, calcium phosphate cement; OCN: osteocalcin; rhBMP-2, recombinant human bone morphogenetic protein-2; rhTGF-β1, recombinant human transforming growth factor-beta 1; Runx-2, runt-related transcription factor 2; Tb.N, trabecular number; Tb.Sp, trabecular separation; Tb.Th, trabecular thickness; ↑: increase/improve, ↓: decrease/inhibit.

**Table 3:** The bone protecting effects of CPC enhanced by proteins.

Enhancer	Characteristics of Enhanced CPC	Type of Study	Type of Cell, Sample, and Animal Model	Outcomes Observed in Animals	Reference
Alkaline-treated silk fibroin	Compressive strength: ↑, setting time: ↓, anti-washout: ↑, injectability: ↑	Ex vivo	Sheep vertebra	Compressive strength: ↑, stiffness: ↑	[17]
		In vitro	MC3T3-E1 cells	No cytotoxicity, good cell morphology	
Lysine	Compressive strength: ↑, apparent porosity: ↑	In vitro	Bone mesenchymal stem cells	ALP: ↑, Runx-2: ↑, COL1: ↑, OCN: ↑	[18]
Arginine-glycine-aspartate	Flexural strength: ↔, elastic modulus: ↔, work of fracture: ↔	In vitro	Human umbilical cord mesenchymal stem cells	Viable cells: ↑, ALP: ↑, COL1: ↑, OCN: ↑, Runx-2: ↑, mineralisation: ↑	[19]
Arginine-glycine-aspartate	Setting time: ↔, flexural strength: ↔, elastic modulus: ↔	In vitro	Human umbilical cord mesenchymal stem cells	Cell density: ↑, ALP: ↑, OCN: ↑, COL1: ↑, mineralisation: ↑	[20]
N-acetyl cysteine loaded silk fibroin	-	Ex vivo	Sheep vertebra	Compressive strength: ↑, stiffness: ↑	[21]
		In vivo	Bone defect at distal femoral metaphysis of male Sprague-Dawley rats	Maximum force: ↑, BV/TV: ↑, remaining material in bone: ↓	
		In vitro	Rat bone marrow mesenchymal stromal cells	ALP: ↑, mineralisation: ↑, Runx-2: ↑, OSX: ↑, OCN: ↑, β-catenin: ↑	
Collagen	Flexural strength: ↑	In vitro	Human umbilical cord mesenchymal stem cells	Mineral nodules: ↑, extracellular matrix formation: ↑, cell number: ↑, ALP: ↑, OCN: ↑, COL1: ↑, Runx-2: ↑, mineralisation: ↑	[22]
Collagen microsphere	-	In vivo	Bone defect at femoral condyles of female New Zealand rabbits	New bone formation: ↑	[23]
Gelatine	Initial and final setting time: ↓, compressive strength: ↑, elastic displacement: ↑	In vitro	Human osteosarcoma (G-292) cells	Cell number: ↑, ALP: ↑	[24]
Gelatine	Setting time: ↓, compressive strength: ↑	In vitro	Human osteoblast-like (MG63) cells	Cell proliferation: ↑, ALP: ↑, type 1 pro-collagen: ↑, TGF-β1: ↑	[25]
Elastin-like polypeptide	Micro-hardness: ↑, compressive strength: ↑, initial and final setting time: ↑, anti-washout, denser microstructure with fewer pores, crystallite formation: ↑	In vitro	NIH3T3 cells	Viable cells, normal cell morphology, normal spreading pattern, normal cell distribution, no nuclear condensation in cells	[26]
CGRP	Pore size distribution: ↔, compressive strength: ↔	In vitro	Rat bone marrow mesenchymal stromal cells	Cell proliferation: ↑, ALP: ↑, BMP-2: ↑, osteonectin: ↑, Runx-2: ↑	[27]
Bone sialoprotein	-	In vitro	Human primary osteoblasts	Cell number: ↑, ALP: ↔, OPN: ↔, OSX: ↑, Runx-2: ↔, osteonectin: ↑	[28]
Bone sialoprotein	-	In vivo	Bone defect at femoral condyles of male Wistar rats	BV/TV: ↔, bone ingrowth: ↔	[29]

Abbreviations: ALP, alkaline phosphatase; BMP-2, bone morphogenetic protein-2; BV/TV, bone volume/total volume; CGRP, calcitonin gene-related peptide; COL1, type I collagen; CPC, calcium phosphate cement; OCN, osteocalcin; OPN, osteopontin; OSX, osterix; Runx-2, runt-related transcription factor 2; ↑, increase/improve; ↓, decrease/inhibit; ↔, no change.

**Table 4:** The bone protecting effects of CPC enhanced by polysaccharides.

Enhancer	Characteristics of Enhanced CPC	Type of Study	Type of Cell, Sample, and Animal Model	Outcomes Observed in Animals	Reference
Bacterial cellulose	Thermal stability: ↑, compressive strength: ↑	In vitro	MC3T3-E1 cells	Cell growth and proliferation: ↑	[50]
Chitosan	-	In vitro	Osteoblastic cells	ALP: ↑	[51]
Chitosan	Flexural strength: ↑, elastic modulus: ↑	In vitro	Rat bone marrow mesenchymal stem cells	ALP: ↑	[52]
Chitosan	Compressive strength: ↑	In vivo	Bone defect at radius of mature dogs	Amount of implant debris: ↓, new bone callus formation: ↑	[53]
Hyaluronic acid	-	In vitro	Osteoblastic cells	ALP: ↑	[51]
Hyaluronic acid	Compressive strength: ↑	In vitro	Human bone marrow mesenchymal stromal cells	ALP: ↑, OPN: ↑, Runx-2: ↑	[54]
		In vivo	Bone defect at metaphyseal region of medial tibia in female Sprague-Dawley rats	BV/TV: ↑, Tb.Pf: ↑, BMD: ↑, bone and vessel formation: ↑, mineralisation: ↑, OCN: ↑, COL1: ↑, BMP-2: ↑	
Alginate	Injectability: ↑, cohesion: ↑, compressive strength: ↑, Young’s modulus: ↑, setting time: ↔	In vitro	Human osteoblast-like cells	Viable cells: ↑	[55]
Alginate hydrogel microbeads	Flexural strength: ↑, work of failure: ↑	In vitro	Human umbilical cord mesenchymal stem cells	ALP: ↑, OCN: ↑, COL1: ↑, OSX: ↑	[56]

Abbreviations: ALP, alkaline phosphatase; BMD, bone mineral density; BMP-2, bone morphogenetic protein-2; BV/TV, bone volume/total volume; COL1, type I collagen; CPC, calcium phosphate cement; OCN, osteocalcin; OPN, osteopontin; OSX, osterix; Runx-2, Runt-related transcription factor 2; Tb.Pf, trabecular pattern factor; ↑, increase/improve; ↓, decrease/inhibit; ↔, no change.

**Table 5:** The bone protecting effects of CPC enhanced by blood components.

Enhancer	Characteristics of Enhanced CPC	Type of Study	Type of Cell, Sample, and Animal Model	Outcomes Observed in Animals	Reference
Blood composite	Initial setting time: ↑, compressive strength: ↔, stiffness: ↓	In vivo	Bone defect at distal femoral end of adult female New Zealand white rabbits	Degradation rate: ↑, new bone formation: ↑	[68]
Platelet-rich plasma	No disintegration of paste consistency, setting time: ↓, compressive strength: ↔	In vitro	MC3T3-E1 cells	No cytotoxic effect, cell proliferation: ↑, cell-to-cement interaction: ↑	[69]
		In vivo	Bone defect at femoral head of male New Zealand white rabbits	Residuary material: ↓, BV/TV: ↑	
Platelet-rich plasma	-	In vivo	Bone defect at distal 1/3 of the caudal vertebra body in ovariectomised female Sprague-Dawley rats	BV/TV: ↑, Tb.Th: ↑, Tb.N: ↑, Tb.Sp: ↓, BMD: ↑, new bone formation: ↑, osteogenesis grade: ↑	[1]
Platelet-rich plasma	-	In vitro	Progenitor bone cells	ALP: ↑, diametral tensile strength: ↔	[70]
		In vivo	Bone defect at femur of rabbits	New trabecular bone formation: ↑, breakdown of bulk dense implants into pieces was observed.	
Fibrin glue	-	In vivo	Bone defect at femoral condyles of male New Zealand white rabbits	Compressive strength: ↑, elastic modulus: ↑, new bone formation: ↑	[71]

Abbreviations: ALP, alkaline phosphatase; BMD, bone mineral density; BV/TV, bone volume/total volume; CPC, calcium phosphate cement; Tb.N, trabecular number; Tb.Sp, trabecular separation; Tb.Th, trabecular thickness; ↑, increase/improve; ↓, decrease/inhibit; ↔, no change.

**Table 6:** The bone protecting effects of CPC enhanced by the combination of biological enhancers.

Enhancer	Characteristics of Enhanced CPC	Type of Study	Type of Cell, Sample, and Animal Model	Outcomes Observed in Animals	Reference
(A) Combination of BMP-2 and other enhancers					
BMP-2-loaded collagen	Setting time: ↓, compressive strength: ↑, disintegration or degree of cohesion: ↔	In vitro	MC3T3-E1 cells	Cell viability: ↑, cell density: ↑	[78]
		In vivo	Bone defect at the parietal part of femur of New Zealand white rabbits	New bone tissue formation: ↑, degradation of material: ↑	
BMP-2 loaded gelatine microsphere	-	In vivo	Bone defect at lumbar vertebrae of female ovariectomised goats	Pushout value: ↑, bone mineralisation: ↑	[79]
rhBMP-2 loaded silk fibroin	-	In vivo	Interbody defect at midpoint of disc space of mature sheep	Stiffness: ↑, BV/TV: ↑, ceramic residue volume: ↓	[80]
(B) Combination of protein and polysaccharide					
Chitosan with arginine-glycine-aspartate motif	Setting time: ↔, flexural strength: ↑, elastic modulus: ↔, work of fracture: ↑	In vitro	Human embryonic stem cell-derived mesenchymal stem cells	Percentage of live cells: ↑, cell density: ↑, OCN: ↑, COL1: ↑, mineralisation: ↑	[81]
Chitosan with arginine-glycine-aspartate motif	Flexural strength: ↑	In vitro	Mouse pluripotent C3H10T1/2(C3) cells	Cell number: ↑, cell proliferation: ↑, ALP: ↑,	[82]
		In vivo	Bone defect at femoral condyles of New Zealand white rabbits	New bone volume: ↑	
(C) Combination of two different polysaccharides					
Chitosan-alginate complex	Initial and final setting time: ↓, no disintegration, compressive strength: ↑	In vivo	Bone defect at femoral head of male New Zealand white rabbits	New bone formation: ↑, implant remaining: ↓	[83]
Alginate-chitosan microencapsulated MC3T3-E1 cells	Setting time: ↔, compressive strength: ↓	In vivo	BALB/c nude mice	Scaffold remaining: ↓, lamellar-bone-like mineral structure: ↑, newly formed collagen: ↑, mineralisation rate: ↑	[84]

Abbreviations: ALP, alkaline phosphatase; BMP-2, bone morphogenetic protein-2; BV/TV, bone volume/total volume; COL1, type I collagen; CPC, calcium phosphate cement; OCN, osteocalcin; rhBMP-2, recombinant human bone morphogenetic protein-2; Runx-2, Runt-related transcription factor 2; ↑, increase/improve; ↓, decrease/inhibit; ↔, no change.

**Table 7:** Summary on the characteristics of CPC after incorporation of biological enhancers.

	Bone-Related Transcription Factors	Proteins	Polysaccharides	Blood Components	Bone-Related Transcription Factors + Proteins	Proteins + Polysaccharide	Polysaccharide + Polysaccharide
Physical properties	-	↑ injectability ↑ anti-washout ↓ setting time (except for elastin-like polypeptide) Pore size: lysine increased, elastin-like polypeptide reduced but CGRP has no change in porosity	↑ injectability ↑ thermal stability ↑ cohesion No change in setting time	Whole blood increased but PRP reduced setting time	↓ setting time No change in disintegration or cohesion	No change in setting time	↓ setting time No disintegration
Mechanical properties	↑ compressive strength	↑ compressive strength (except for CGRP) ↑ flexural strength ↑ elasticity ↑ work of fracture ↑ micro-hardness	↑ compressive strength ↑ flexural strength ↑ elasticity ↑ work of fracture	No improvement in compressive strength ↓ stiffness	↑ compressive strength	↑ flexural strength No change in elasticity ↑ work of fracture	↑ compressive strength
Biological properties	↑ osteogenesis ↑ bone density, microstructure, and strength	No cytotoxicity ↑ osteogenesis (except bone sialoprotein) ↑ bone microstructure and strength	No cytotoxicity ↑ osteogenesis ↑ bone density and microstructure	No cytotoxicity ↑ osteogenesis ↑ bone density and microstructure	No cytotoxicity ↑ osteogenesis ↑ bone microstructure and strength	No cytotoxicity ↑ osteogenesis ↑ bone microstructure	↑ bone formation and mineralisation

Abbreviations: CGRP, calcitonin gene-related peptide; CPC, calcium phosphate cement; PRP, ↑, increase/improve; ↓, decrease/inhibit.

## Data Availability

Not applicable.
